# Updates on the Taxonomy of Mucorales with an Emphasis on Clinically Important Taxa

**DOI:** 10.3390/jof5040106

**Published:** 2019-11-14

**Authors:** Grit Walther, Lysett Wagner, Oliver Kurzai

**Affiliations:** 1 German National Reference Center for Invasive Fungal Infections, Leibniz Institute for Natural Product Research and Infection Biology – Hans Knöll Institute, 07745 Jena, Germany; Lysett.Wagner@hki-jena.de (L.W.); okurzai@hygiene.uni-wuerzburg.de (O.K.); 2 Institute for Hygiene and Microbiology, University of Würzburg, 97080 Würzburg, Germany

**Keywords:** Mucorales, taxonomy, pathogens, identification, ecology, *Circinella*, *Lichtheimia*, *Mucor*, *Rhizomucor*, Rhizopus

## Abstract

Fungi of the order Mucorales colonize all kinds of wet, organic materials and represent a permanent part of the human environment. They are economically important as fermenting agents of soybean products and producers of enzymes, but also as plant parasites and spoilage organisms. Several taxa cause life-threatening infections, predominantly in patients with impaired immunity. The order Mucorales has now been assigned to the phylum Mucoromycota and is comprised of 261 species in 55 genera. Of these accepted species, 38 have been reported to cause infections in humans, as a clinical entity known as mucormycosis. Due to molecular phylogenetic studies, the taxonomy of the order has changed widely during the last years. Characteristics such as homothallism, the shape of the suspensors, or the formation of sporangiola are shown to be not taxonomically relevant. Several genera including *Absidia*, *Backusella*, *Circinella*, *Mucor*, and *Rhizomucor* have been amended and their revisions are summarized in this review. Medically important species that have been affected by recent changes include *Lichtheimia corymbifera, Mucor circinelloides,* and *Rhizopus microsporus.* The species concept of *Rhizopus arrhizus* (syn. *R. oryzae*) is still a matter of debate. Currently, species identification of the Mucorales is best performed by sequencing of the internal transcribed spacer (ITS) region. Ecologically, the Mucorales represent a diverse group but for the majority of taxa, the ecological role and the geographic distribution remain unknown. Understanding the biology of these opportunistic fungal pathogens is a prerequisite for the prevention of infections, and, consequently, studies on the ecology of the Mucorales are urgently needed.

## 1. Introduction

Members of the order Mucorales represent a permanent part of the human environment, as pioneers on all kinds of wet organic materials and causative agents of rot and spoilage, as well as being ingredients of cheese and soy products [1,2]. The order includes numerous thermotolerant or thermophilic species that are able to grow at human body temperature. Several of these species can cause life-threatening infections (mucormycosis), mostly in patients with impaired immunity [3,4]. On the other hand, mucoralean fungi have been used for centuries to ferment traditional Asian and African food [5] and are utilized for the production of several varieties of European cheese [2]. As producers of a broad spectrum of enzymes, Mucorales are used in biotechnology for the biotransformation of several medically and pharmaceutically important compounds such as steroids and terpenoids [1,2,6]. Our understanding of Mucorales biology has severely suffered from a largely unresolved taxonomy. Even in the medical setting, these fungi are often not identified to the species level. However, significant progress in understanding Mucorales taxonomy has been made in recent years. Here we address essential changes in the taxonomy of this medically and economically important group of fungi, with an emphasis on medially-relevant taxa.

## 2. Higher-Level Classification and Delimitation of the Mucorales

**Figure 1:**
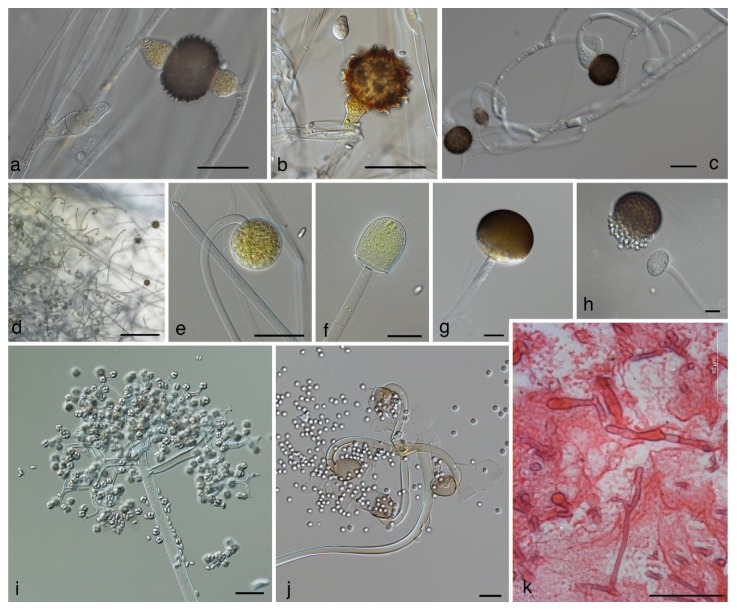
Morphology of the Mucorales. (**a**) Zygospore with equal suspensors of *Mucor endophyticus* CBS 385.95; (**b**) azygospore of *Mucor bainieri* CBS 293.63; (**c**) zygospores with unequal suspensors of *Mucor multiplex* (syn. *Zygorhynchus multiplex*) CBS 110662; (**d**) top view on a young mycelium with transitorily recurved sporangiophores; (**e**) recurved sporangiophore and (**f**) columella of *Backusella recurva* CBS 318.52; (**g**) sporangium with circumscissile zone of dehiscence and (**h**) discharged sporangium of *Pilaira anomla* CBS 699.71; (**i**) sporangiola-bearing complex sporophore of *Thamnidium elegans* CBS 341.55; (**j**) circinate sporangiophore branches with columellae and detached sporangiospores of *Circinella umbellata*; (**k**) hyphae of *Rhizopus microsporus* in human lung tissue. Scale bars = 50 µm except of d = 500 µm.

If members of the Mucorales reproduce sexually, they reproduce by zygospores. These are thick-walled, pigmented, and often ornamented zygotes (Figure 1a) that are formed by the fusion of two differentiated hyphal ends, including fusion of the cytoplasm and nuclei. In previous, morphology-based classifications, fungi reproducing by zygospores—for which we will use the informal term “zygomycetes” for the rest of this review—were classified in the phylum Zygomycota [7]. However, the phylum Zygomycota was abandoned because it was not supported in molecular phylogenies that included a higher number of loci and taxa [8]. As a result of these analyses, the subphylum Mucoromycotina, including the Mucorales, the Endogonales, and the Morteriellales, was treated as incertae sedis—a taxon that is not assigned to the next higher-level taxon (the phylum in this case). Erroneously, in a publication widely noticed by medical mycologists [9], the Mucoromycotina were described as a subphylum of the Glomeromycota, a fact that resulted in numerous incorrect citations [10,11].

**Figure 2:**
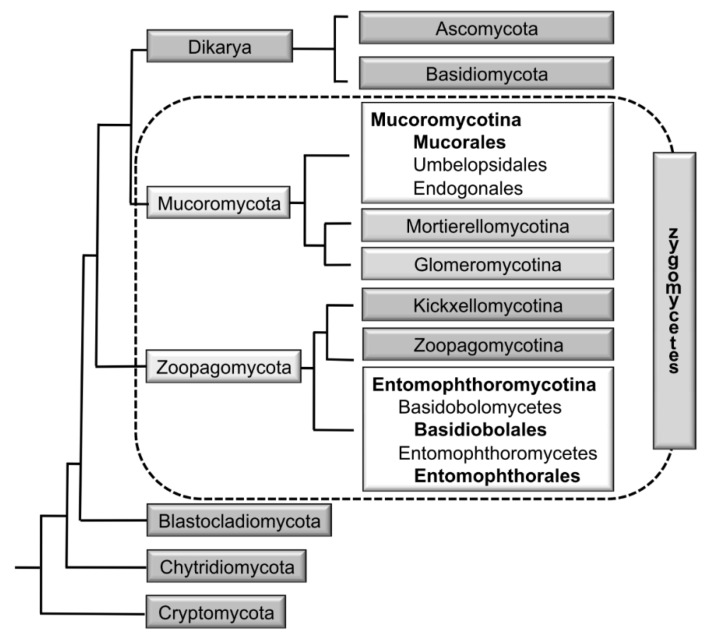
Classification of zygomycete fungi including the Mucorales based on Spatafora et al. [12]. Bold printed taxa contain pathogenic species.

Recently, Spatafora et al. [12] analysed the genomes of 46 taxa, including 25 zygomycetes. In their phylogenetic analyses of 192 proteins, the zygomycetes were comprised of two novel clades with a paraphyletic relationship (Figure 2). For each of the two clades a phylum was circumscribed: the Mucoromycota and the Zoopagomycota. These analyses revealed the Mucoromycota as a sister clade to the Asco- and Basidiomycota and the non-existence of a zygomycetes clade. The Mucoromycota are mainly associated with plants and comprised of three subphyla: (1) the arbuscular mycorrhizae-forming Glomeromycotina, (2) the Mortierellomycotina, that are frequently root endophytes [13], and (3) the Mucoromycotina, consisting of the orders Mucorales, Umbelopsidales, and Endogonales. The Mucorales include many decomposers of dead plant material and parasites of plants [1], the Umbelopsidales comprise endophytes and saprobes of forest soils, and the Endogonales are saprobes or form ectomycorrhizas. In contrast, the Zoopagomycota are either saprobes or parasites of animals and other fungi. They include the saprotrophic or insect pathogenic Entomophthorales, with the opportunistic genera *Basidiobolus* (Figure 1d,e) and *Conidiobolus* (Figure 1f,g) [12].

As a consequence of the phylogenetic distance and taxonomic separation of the Mucorales and the Entomophthorales, the term “zygomycosis’’ (infections caused by members of the Zygomycota) was abandoned and instead the term ‘‘mucormycosis’’ was used for infections caused by members of the Mucorales and “entomophthoromycosis” was used for infections caused by members of the Entomophthorales [9]. Mucormycoses are generally angioinvasive, have an acute course, and affect predominantly immunocompromised individuals, whereas entomophthoromycoses are usually subcutaneous, show a chronic course, and mostly affect immunocompetent individuals. In histopathologic sections, mucormycoses and entomophthoromycoses are very similar, showing broad, belt-like, non- or rarely-septated hyphae (Figure 1k). However, it has been suggested that both entities can be differentiated in hematoxylin–eosin stained sections, where only the hyphae of the Entomophthorales are surrounded by eosinophilic sleeves [9].

**Figure 3:**
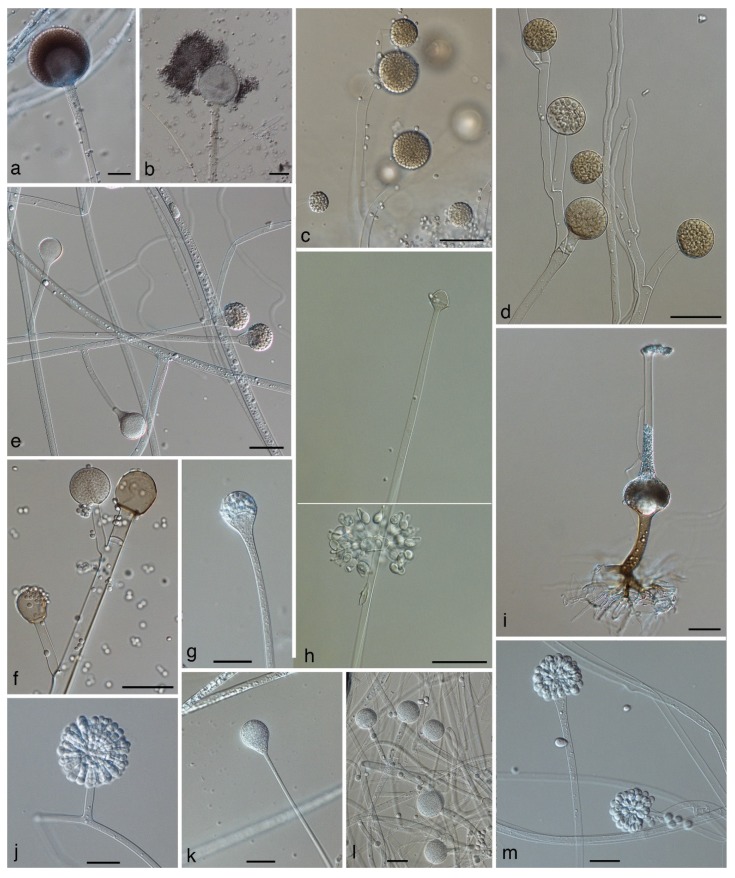
Morphology of the opportunistic members of the Mucorales. (**a**) Sporangiophore with sporangium and indistinct apophysis and (**b**) with columella and detached sporangiospores of *Rhizopus arrhizus* JMRC:NRZ:1217; (**c**) sympodially branched sporangiophore of *Mucor circinelloides* NRZ-2019-468; (**d**) sympodially branched sporangiophore of *Mucor ramosissimus* CBS 135.65; (**e**) sporangiophore with columellae and detached sporangiospores of *Lichtheimia ramosa* NRZ-2019-435; (**f**) sporangiophore with columellae and detached sporangiospores of *Rhizomucor pusillus* JMRC:NRZ:0496; (**g**,**k**) sporangiophores of *Apophysomyces variabilis*; (**h**) sporophore with sporangiola and columella at the place of the former sporangium of *Thamnostylum lucknowense* JMRC:SF:00845; (**i**) sporophore of *Saksenaea* sp.; (**j**) sporophore with merosporangia of *Syncephalastrum racemosum* CBS 302.65; (**l**) young sporangiophore of *Actinomucor elegans* CBS 111556; (**m**) sporophores with single-spored sporangiola of *Cunninghamella bertholletiae.* Scale bars = 50 µm.

Mucorales produce their uni-celled asexual spores (sporangiospores) endogenously, i.e., inside specialized cells. In contrast to the formation of conidia, cell walls of the sporangiospores are formed without the involvement of pre-existing cell walls. The spore forming specialized cells are named according to their shape and the number of spores they contain: sporangia (Figure 1e,g, Figure 3a,c,d,g) are globose cells containing a high (uncountable) number of sporangiospores, sporangiola (Figure 1i, Figure 3h,m) are globose cells containing one to a countable number of sporangiospores, and merosporangia (Figure 3j) are elongated cells containing one to a countable number of sporangiospores. In the past, uni-spored sporangiola (Figure 3m) were called “conidia”, but this term should be avoided. Conidia are, by definition, exogenously formed, while the conidia-like structures in the Mucorales are single sporangiospores formed inside a sporangiolum recognizable by two adjacent cell walls: the inner wall representing the wall of the sporangiospore, and the outer wall representing the wall of the sporangiolum [1]. In contrast, the opportunistic genera *Basidiobolus* and *Conidiobolus* of the Zoopagomycota reproduce asexually by true conidia that are forcibly discharged (Figure 1d,f) [1].

The main characterizing feature of the order Mucorales used to be the columella (Figure 1f)—a sterile central vesicle inside the sporangium (Figure 1e,g). The genus *Umbelopsis* was previously classified in the Mucorales, although it develops much smaller columellae than other members of the Mucorales. As a result of the genome analysis performed by Spatafora et al. [12], the genus *Umbelopsis* is now classified in its own order, the Umbelopsidales, and consequently columellae are formed in two orders now: the Mucorales—with pronounced columellae (with some exceptions), and the Umbelopsidales with inconspicuous columellae. Members of the Mucorales are usually fast-growing. As is typical for zygomycetes, they grow in coenocytic hyphae that do not have septae at regular distances and include a high number of nuclei. Septae are usually formed to delimit reproductive structures or swollen parts. In tissue infection, they may produce septae but not at regular distances as in ascomycetous fungi (Figure 1k).

The first molecular phylogenies based on two phylogenetic markers (fragment of coding or non-coding DNA used in phylogenetic reconstructions) [14,15] resulted in the revision of the family concepts of the Mucorales [16] due to polyphyly (descent from more than one ancestral group) of the sporangiola-forming taxa previously united in the Thamnidiaceae. By using an extended set of species and three loci, Hoffmann et al. [17] further enhanced the family concept and recognized 13 families exclusive of the Umbelopsidaceae that now belong to a separate order [12]. The main changes in the family concept were as follows: *Absidia* was assigned to the Cunninghamellaceae, and new families were erected to incorporate *Backusella* (Backusellaceae), *Lentamyces* (Lentamycetaceae), and *Rhizopus*, *Syzygites,* and *Sporodiniella* (Rhizopodaceae). The Lichtheimiaceae were extended by the genera *Circinella*, *Dichotomocladium*, *Fennellomyces Phascolomyces*, *Rhizomucor*, *Thamnostylum*, *Thermomucor*, and *Zychaea*.

## 3. Lower-Level Classification: Molecular Taxonomy and Re-Evaluated Criteria for Genus and Species Delimitation

Members of the Mucorales seem to have a dynamic organisation of their genomes. The genome of *Rhizopus arrhizus* (syn. *R. oryzae*), and also most likely those of *Mucor circinelloides* and *Phycomyces blakesleeanus* have undergone whole genome duplication [18,19], while in *Lichtheimia corymbifera* only single genes were duplicated [20]. Several markers that are traditionally used in the phylogeny of higher fungi cannot be applied to Mucorales due to the presence of paralogous genes [21,22,23]. As a result, the number of studies based on at least two, unlinked markers that meet the criteria of phylogenetic species recognition by genealogical concordance is comparatively low [24] (s. Table 1). Appropriate markers for the opportunistic genera *Apophysomyces*, *Lichtheimia*, *Mucor,* and *Rhizopus* are limited. In *Lichtheimia,* all the established phylogenetic markers, such as the partial genes of actin, beta-tubulin, the first (*rpbq1*) and the second largest subunit (*rpb2*) of RNA polymerase II, and the translation elongation factor-1 alpha (*tef*) possess paralogs [25]. In *Mucor*, the genes of actin, *tef*, beta-tubulin, and calmodulin are also not single-copy genes [23]. In *Rhizopus arrhizus*, *tef* has paralogs, but the polymorphisms are restricted to the third position of the triplet codon so that *tef* could be used as marker if these positions were excluded from the analyses [26].

Due to this lack of additional markers, most descriptions of new species are still based on a single locus, usually ITS sequence alone, or combined with LSU sequences [27,38,49,50,51,52], for which genealogical concordance phylogenetic species recognition cannot be applied. This practice can be acceptable in taxa with well-defined molecular taxonomy and species variabilities. However, in unrevised genera lacking molecular species concepts (e.g., *Absidia*), the description of new species needs to be based on a comprehensive molecular and phenotypic study of the sibling species as well. 

Some of the recent descriptions of new species and new genera [53,54,55,56] are only based on morphological features. Considering the morphological plasticity of the Mucorales shown by numerous reclassifications of species based on molecular data [22], it is our opinion that new species descriptions should include molecular data. This is especially important because a subsequent molecular evaluation of species is not always possible, as several strain collections do not provide strains outside their country or overseas (personal observation).

When molecular phylogenies were available, it became apparent that phenotypic characteristics traditionally used in mucoralean taxonomy were not always taxonomically informative. In the past, the formation of sporangiola or a combination of sporangiola and sporangia has been used to define new genera, e.g., *Backusella* [57] and *Isomucor* [21]. However, according to molecular phylogenies [14,15,17,22], sporangiola-forming species appear intermittantly among *Mucor* species. In the morphologically and molecularly well-defined genus *Backusella,* some, but not all, species produce sporangiola in addition to sporangia. Likewise, in the *Mucor circinelloides* complex [23], only two out of 14 species form sporangiola. Thus, the formation of sporangiola could either be a plesiomorphic character in the Mucorales that is genetically fixed, but not revealed in all species, or one that has evolved several times in surprisingly short phylogenetic distances, and often in connection to dung as habitat. Distinctly curved (circinate) sporangiophores, combined with persistent walls of the sporangia, occur not only in the genus *Circinella* (Figure 1j), but also in two different lineages of the genus *Mucor* [22,23].

Another feature that turned out not to be taxonomically informative is the shape of the suspensors (supporting hyphae of the zygospores, Figure 1a,c). The genus *Zygorhynchus* was described by Vuillemin [58] to accommodate Mucor-like species with unequally shaped suspensors. Typically, one suspensor is hypha-like and the other suspensor is swollen (Figure 1c). In all *Zygorhynchus* species, this feature was combined with the formation of zygospores on the same mycelium (homothallism) and on the same hypha (*Zygorhynchus* pattern of zygospore formation). The features of unequally shaped suspensors, zygospore formation according to the *Zygorhynchus* pattern, and homothallism are linked for unknown reasons, but this combination of traits is convergent within *Mucor.* Therefore, all *Zygorhynchus* species were recombined in *Mucor* [53]. Recently the genus *Zygambella* was erected based solely on a morphological description [53]. The homothallic *Zygambella* strongly resembles *Rhizopodopsis* in having umbellate, pigmented sporangiophores, but differs from *Rhizopodopsis* only by its unequal suspensors. Judging from the *Zygorhynchus* example, *Zygambella* is likely to be only a homothallic member of *Rhizopodopsis*.

In addition to morphological characters, the formation of zygospores in crosses of two strains has been traditionally used to define biological species boundaries [59,60,61]. Crosses between different but closely related species were assumed to result in the formation of azygospores [62], or lead to the production of zygospores that differ in size, colour, ornamentation, or number from intraspecific zygospores [25,63]. Azygospores resemble zygospores. They can be either formed on a single suspensor (Figure 1b) or on two suspensors, where the septum between the differentiated hyphal tips (gametangia) remains and no plasmogamy takes place [62]. However, Wagner et al. [23] showed for the *Mucor circinelloides* relationship that the differences between intra- and interspecific zygospores can be inconspicuous and only related to the size of the zygospores and the height of their ornamentation. Consequently, the differentiation between intra- and interspecific zygospores might require elaborate studies, including numerous crosses. The simple presence of zygospores between two strains cannot be used as criterion of conspecificity.

## 4. Mucoralean Genera and Species with Changed Taxonomic Concepts

The Mucorales in the sense of Spatafora et al. [12] comprise 261 species in 55 genera [64,65] (Table 2). Twenty-eight of the species have been described since 2000. Thirty-eight out of these 261 species have been reported to be involved in human infections (Table 3). Genera including opportunistic species are *Actinomucor* (Figure 3l), *Apophysomyces* (Figure 3g,k), *Cokeromyces*, *Cunninghamella* (Figure 3m), *Lichtheimia* (Figure 3e), *Mucor* (Figure 3c,d), *Rhizomucor* (Figure 3f), *Rhizopus* (Figure 3a,b), *Saksenaea* (Figure 3i), *Syncephalastrum* (Figure 3j), and *Thamnostylum* (Figure 3h). Here we detail the genera and species where the taxonomic concept has been updated.

### 4.1. Absidia, Lentamyces, and Lichtheimia (Figure 3e)

Originally the genus *Absidia* united species with pyriform sporangia and a distinct apophysis (a dilatation of the sporangiophore underneath the sporangium, Figure 3e) and hyaline, branched sporangiophores. Phylogenetic and physiological studies showed that Absidia-like fungi represent three separate lineages [79]: (1) the mesophilic genus *Absidia* sensu stricto that forms zygospores protected by long appendages of the suspensors; (2) the mycoparasitic genus *Lentamyces* that does not grow at temperatures above 30 °C; and (3) the thermotolerant and opportunistic genus *Lichtheimia* that produces zygospores with equatorial rings and suspensors without appendages. *Lichtheimia* was first named *Mycocladus*, typified by *Mycocladus verticillatus* [79]. However, the type material of that species did not belong to the thermotolerant species, but possibly represented a mixed culture of *Absidia* s. str. and *Lentamyces* [80]. As a consequence, the thermotolerant species were renamed again in the oldest available genus name, *Lichtheimia* [80]. Garcia-Hermoso et al. [33] showed that the clinical isolates in their study actually belonged to two species, *Lichtheimia corymbifera* and *Lichtheimia ramosa,* which were treated as synonyms previously. Alastruey-Izquierdo et al. [25] revised the whole genus and recognized five species in *Lichtheimia*: *L. corymbifera*, *L. hyalospora*, *L. ornata*, *L. ramosa*, and *L. sphaerocystis*. Later a sixth species, *L. brasiliensis,* was described [81]. Only *L. corymbifera*, *L. ornata*, and *L. ramosa* are reported to cause human infections [25,82]. The clinical importance of *Lichtheimia* spp. depends on the geographical region. In Europe and Africa *Lichtheimia* species are the second most frequently reported aetiological agents of mucormycoses behind *Rhizopus* spp. while in America the number of cases is rather low. Most cases caused by *Lichtheimia* spp. show a cutaneous or pulmonary manifestation but also rhino-orbital-cerebral and disseminated infections occur [83].

*Lichtheimia corymbifera* was described to form subglobose to broadly ellipsoidal spores, while *L. ramosa* was thought to develop ellipsoidal to cylindrical spores only. When Nottebrock et al. [84] crossed strains of the two species identified by the shape of their spores, zygospores were produced, Subsequently, the two species were treated as synonyms until Garcia-Hermoso et al. [33] showed them to be distinct species. Alastruey-Izquierdo et al. [25] found strains of both species with an intermediary spore shape, such that misidentified strains could be responsible for the positive mating results of Nottebrock et al. [84]. The clinically relevant *Lichtheimia* species can be distinguished phenotypically. *Lichtheimia ramosa* has a higher growth rate at 43 °C than *L. corymbifera* and *L. ornata*. *Lichtheimia ornata* can be distinguished from *L. corymbifera* by its densely packed giant cells (large, irregularly shaped cells) formed on yeast extract agar. Important for the differentiation of *Absidia* and *Lichtheimia* are the different maximum growth temperatures and the formation of a septum directly underneath the sporangium (subsporangial septum) in *Absidia* but (with rare exceptions) not in *Lichtheimia* [25].

### 4.2. Actinomucor elegans (Figure 3l)

Only a few cases of *Actinomucor* infections have been reported [83,85,86]. The genus *Actinomucor* consists only of a single species, *Actinomucor elegans,* for which three varieties are described: var. *elegans*, var. *meitauzae*, and var. *kuwaitiensis.* These varieties differ in shape, size, and ornamentation of the sporangiospores [87,88] and their growth on Czapek’s agar [89]. However, the varieties are not detected in ITS-based phylogenetic analysis, suggesting that these differences might not be phylogenetically relevant [22].

### 4.3. Backusella (Figure 1d–f)

The genus *Backusella* has not been involved in human infections. It was erected by Ellis and Hesseltine [57] for Mucor-like species producing sporangiola on side branches of the main sporangiophore, in addition to sporangia. Molecular phylogenetic studies based on LSU and ITS sequences revealed a clade of *Backusella* and *Mucor* species characterized by transitorily recurved sporangiophores (Figure 1d,e), i.e., young sporangiophores that are curved, but become upright during the maturation of the sporangium. As a consequence, several *Mucor* species were transferred to *Backusella* [22]. Based on the same study, *Backusella ctenidius* was assigned to *Mucor*.

### 4.4. Circinella (Figure 1j)

The genus *Circinella* has not been reported to cause infections. It was erected beside *Mucor* in order to accommodate species with strikingly circinate sporangiophores and sporangia with persistent walls [90]. Sequence analyses revealed that *Circinella* was polyphyletic, with the main group, including the type species *Circinella umbellata,* positioned in the Lichtheimiaceae [17,22]. *Circinella rigida* turned out to belong to the genus *Mucor* and was renamed as *Mucor durus* [22]. Strains formerly assigned to *Circinella simplex* that produce angular spores and secondary branches of the sporangiophores were placed in the *Mucor* clade in molecular phylogenetic analyses. Van Tieghem [91], however, described and illustrated *C. simplex* with globose sporangiospores and sporangiophores without secondary branches. While *C. simplex* was isolated from dog droppings in France, all angular-spored strains originate from the tropics. Consequently, the angular-spored strains were described as a separate species, *Mucor circinatus* [35]. A recent taxonomic study of *Circinella* [55] is only based on morphology and the two, newly described species *C. nodulosa* and *C. ramosa* need to be evaluated on molecular grounds.

### 4.5. Mucor (Including Zygorhynchus) (Figure 3c,d)

*Mucor* belongs to the main genera causing mucormycoses [83]. To date, 12 species are known to be involved in infections [36]. Members of the genus predominantly cause cutaneous infections but also disseminated and gastrointestinal manifestations have been reported [2,36]. However, the clinical importance of the genus is still not clear because the causative agents of infections are only morphologically identified to the genus level in most cases [83] and might in fact represent the morphologically similar *Rhizomucor* or *Lichtheimia* species.

The genus *Mucor* is currently made up of 76 accepted species and is by far the largest genus in the Mucorales. Several molecular studies revealed the polyphyly of *Mucor* [14,15,17,22]. *Mucor* was previously characterized by the formation of sporangia and equally shaped suspensors, as well as the absence of apophysis, rhizoids (root-like hyphae), and sporangiola. Recently, it was shown that *Mucor* species are able to form rhizoids [22,23]. This explains the misclassification of several *Mucor* species in the genus *Rhizomucor* due to the formation of rhizoids in culture (for details see *Rhizomucor*). Sequence analyses also revealed that taxa with an apophysis, such as *Mucor durus* (syn. *Circinella rigida*), and with sporangiola, such as *Mucor ctenidius* (syn. *Backusella ctenidia*), belong to the genus *Mucor* [22]. As mentioned before, all *Zygorhynchus* species (Figure 1c) were transferred to *Mucor* [22].

On the basis of morphology and mating experiments, the most clinically relevant *Mucor* species, *M. circinelloides,* used to be divided in four formae: f. *circinelloides*, f. *griseocyanus*, f. *janssenii,* and f. *lusitanicus* [92]. Through the use of multi-locus analyses of five different markers, morphological traits and mating experiments, a recent study showed that the former formae represent, in fact, one or two separate species (Table 3) [23]. *Mucor velutinosus* [46] was supported as a sibling species of *M. janssenii* and five new species were described. Strains that were, in a previous study, misleadingly assigned to *M. fragilis,* are now assigned to *M. variicolumellatus* [22] because *M. fragilis* is related to *Mucor hiemalis*, according to the original description and illustration [92]. *Mucor ellipsoideus* [71] was synonymised with *M. ardhlaengiktus* due to their similar ITS sequences [22].

Based on case reports or strain source information [4,22,23,37,70], the following *Mucor* species are potentially involved in human infections (Table 3): *M. amphibiorum, M. ardhlaengiktus* (syn. *M. ellipsoideus*), *M. circinelloides*, *M. griseocyanus*, *M. indicus, M. irregularis* (syn. *Rhizomucor variabilis*), *M. janssenii*, *M. lusitanicus*, *M. racemosus*, *M. ramosissimus, M. variicolumellatus*, and *M. velutinosus*. *Mucor amphibiorum* is known as a pathogen of amphibians, but a single human isolate has been reported [22]. The involvement of *M. racemosus* in invasive human infections is doubtful because it is morphologically similar to *M. circinelloides*, it does not grow at temperatures above 34 °C and there has been no DNA-based report of the species from invasive infections [23]. The only molecularly-verified reports of this species refer to superficial infections [22]. *Mucor griseocyanus* has not been described from invasive human infections, but was isolated from nails [23]. Reports on *M. hiemalis* likely refer to its sibling species, *M. irregularis* (syn. *Rhizomucor variabilis*) [93] as *M. hiemalis* is not able to grow at temperatures higher than 30 °C [59]. All case reports of this species are based solely on morphology and were published before *Mucor irregularis* was described [71]. In agreement with the typical clinical picture of *M. irregularis* infections, the majority of these reports describe chronic cutaneous infections of healthy individuals [93].

### 4.6. Rhizomucor (Figure 3f)

*Rhizomucor* species cause about 5% of the mucormycoses worldwide ([83]; the percentage given here is reduced by the cases of *Rhizomucor variabililis* because this is now transferred to *Mucor irregularis*). *Rhizomucor* species mainly cause pulmonary infections followed by disseminated, cuteanous, and rhino–orbital–cerebral manifestations [83].

All mesophilic species of *Rhizomucor,* including *Rm. chlamydosporus*, *Rm. endophyticus*, *Rm. regularior* (syn. *Rm. variabilis* var. *regularior*), and *Rm. variabilis* were transferred to *Mucor* (Table 3) based on molecular data [22,71]. In its current classification, *Rhizomucor* only harbours thermophilic species with maximum growth temperatures above 50 °C and minimum growth temperatures below 20 °C. These species, including *Rm. miehei*, *Rm. pusillus*, *Rm. nainitalensis*, and *Rm. pakistanicus,* possess subglobose to short ellipsoidal sporangiospores. There are no strains, sequences, or additional reports available for the latter two species and it cannot be excluded that they represent synonyms of *Rm. pusillus* or *Rm. miehei*.

### 4.7. Rhizopus (Figure 3a,b)

The genus *Rhizopus* is by far the most important causative agent of mucormycoses worldwide and the main cause of rhino–orbital–cerebral infections. Pulmonary, cutaneous, and disseminated disease manifestations are also frequently reported. The majority of cases are caused by two species: most frequently by *R. arrhizus* (syn. *R. oryaze*), followed by *R. microsporus* [83].

The genus *Rhizopus* is characterized by the formation of unbranched, pigmented sporangiophores that arise singly or in whorls and that bear sporangia with an apophysis. Rhizoids are formed opposed to the sporangiophores [94,95]. Phylogenetic analyses of the mushroom parasite, *Syzygites megalocarpus,* and the insect parasite, *Sporodiniella umbellate,* revealed the paraphyletic nature of the genus, with both taxa clustering among *Rhizopus* species [17,22]. The genus *Sporodiniella* shares several traits with *Rhizopus*: they both have pigmented sporangiophores and the sporangia are apophysate. In contrast to *Rhizopus,* the sporangiophores of *Sporodiniella* are arranged in umbels. *Rhizopodopsis* and *Zygambella* [53] also form umbellate sporangiophores with an apophysis and possibly belong in this relationship, but no sequence data are available. In contrast to the insect parasite *Sporodiniella, Rhizopodopsis* was isolated from fallen fruits of the *Elaeagnus* plant and *Zygambella* from soil.

Based on morphological and molecular data [17,22,26,38,44,94,95,96], we accept the following *Rhizopus* species: *R. americanus*, *R. caespitosus*, *R. homothallicus*, *R. lyococcus*, *R. microsporus*, *R. schipperae*, *R. koreanus*, *R. arrhizus* (with the varieties *arrhizus* (syn. *R. oryzae*) and var. *delemar* that are treated as discrete species by some authors (see below)), *R. sexualis*, and *R. stolonifer*. The recently described *R. koreanus* is a sibling species of *R. stolonifer* that differs from the latter only slightly by the size of the columellae [38]. The species was already detected as a cryptic sibling species of *R. stolonifer* based on ITS sequences [22] and probably also by amplified polymorphic DNA (RAPD) patterns [97].

#### 4.7.1. *Rhizopus microsporus*

Three species related to *R. microsporus* that were distinguished only by the shape, the size, and the ornamentation of the sporangiospores were reduced in rank due to positive mating tests [94] and subsequently treated as varieties of *R. microsporus*, namely var. *chinensis*, var. *oligosporus*, and var. *rhizopodiformis.* Later, additional varieties, var. *azygosporus* [95] and var. *tuberosus* [98] were described. Typically, domesticated foodborne strains with reduced and irregular sporulation were assigned to the variety *oligosporus*, while clinical strains were mostly attributed to var. *rhizopodiformis*. However, in sequence based approaches [22,42,44,96] these varieties were not detected. Phylogenetic analyses of three loci (*its*, *act*, *tef*) revealed recombination between two of the three subgroups within *R. microsporus*, with both groups containing all the morphologically-defined varieties [44]. There was no correlation between the phylogenetically-defined subgroups and the varieties. Based on these results, the varieties of *Rhizopus microsporus* were reduced to synonyms [44]. On the genome level, this species shows a large degree of molecular diversity [99].

#### 4.7.2. *Rhizopus arrhizus* (syn. *R. oryzae*) (Figure 3a,b)

There are two pending issues concerning *R. arrhizus*: (1) the correct name: *R. arrhizus* vs. *R. oryzae* and (2) the correct taxonomic rank of *R. delemar (R. delemar* vs. *R. arrhizus* var. *delemar)*. Regarding the first issue, *R. arrhizus* was described by Fischer [100] three years before *R. oryzae* [101]. In contrast to the description of *R. oryzae*, the description of *R. arrhizus* was short, without figures, and no type material was mentioned. As a consequence, the name *R. oryzae* was preferred by most authors. However, Ellis [102] designated an ex-neotype strain for *R. arrhizus*, thus legitimizing the name *R. arrhizus*. Consequently, the name *R. arrhizus* has priority. Because the ex-type strain of *R. oryzae* clusters with the ex-neotype strain of *R. arrhizus*, *R. oryzae* should be treated as synonym of *R. arrhizus* [26].

The second issue does not have such a clear consensus. Variety *arrhizus* produces and accumulates lactic acid in the medium because it possess two slightly differing genes for lactate dehydrogenase (*ldhA* and *ldhB*), while the fumaric and malic acid producing var. *delemar* possess only *ldh*B [41,103]. Based on morphology, Zheng et al. [95] recognized three varieties: var. *arrhizus*, var. *delemar*, and var. *tonkinensis*. However, var. *tonkinensis* was not supported by molecular phylogenetic analyses [26]. According to Zheng et al. [95], the morphological differences between var. *arrhizus* and var. *delemar* were small and quantitative, including the predominant position of swellings in the sporangiophore, the main origin of the sporangiophores (aerial hyphae or stolons = runner hyphae, horizontally growing unbranched hyphae), slight differences in the shape of the columellae, and a larger maximum spore size of var. *delemar*. However, these morphological features are not fully consistent. Strain CBS 395.34 was morphologically identified as var. *arrhizus* [95], but molecular identification related it to var. *delemar* [26]. Additionally, no differences in ecology, distribution, and pathogenicity could be detected between the two varieties. In multi-locus studies, var. *arrhizus* and var. *delemar* were well-recognized without evidence of recombination [26,43]. Although zygospore formation is a rare event in both varieties, zygospores have been observed in crosses between the variety *arrhizus* and variety *delemar* [26,43,94].

Genome sequencing of *R. arrhizus* var. *delemar* revealed a dynamic organization of the genome and indicated an ancestral, whole-genome duplication [18]. Different haploid chromosome numbers were determined in two strains of *R. arrhizus* (at the time considered to belong to different species *R. arrhizus* and *R. niveus*) [104]. On the genome scale, 76 orthologous proteins could not resolve var. *arrhizus* and var. *delemar* [105]. A whole-genome phylogeny based on 1,620,389 single-nucleotide polymorphisms (SNPs) from 13 isolates revealed three clades: one clade containing only var. *arrhizus*, one clade containing only var. *delemar* strains, and the third clade containing strains with mixed ancestry from both var. *arrhizus* and var. *delemar*. No differences in virulence were found between strains of these clades [105]. Gryganskyi et al. [99] analysed 192 orthologous proteins from the genomes of 21 *Rhizopus* strains, including *R. microsporus*, *R. arrhizus* var. *arrhizus* and var. *delemar,* and *R. stolonifer*. They found two well-supported clades for both varieties and a phylogeny based on morphology and physiology that was congruent with the molecular phylogeny. However, in our opinion, these well-supported clades could also represent varieties. Especially if the number of strains is low, strains with mixed ancestry, as detected by Chibucos et al. [105], might not be included. The morphological characters used for their non-molecular phylogeny are in conflict with previous studies: (1) the diameter of the sporangia: the authors in Gryganskyi et al. [99] found them to be 160–240 µm in var. *arrhizus*, and 250–300 µm in var. *delemar,* while Schipper [94] and Zheng et al. [95] described them as up to 176 µm in diameter for both varieties; (2) the shape of the columella: the authors describe the collumellae of var. *arrhizus* as ellipsoidal and those of var. *delemar* as subglobose or conical, while Zheng et al. [95] found the columellae of *R. arrhizus* to be subglobose, hemiglobose, or roundish conical; and (3) the Gryganskyi study found the sporangiospores of var. *arrhizus* to be ridged but not striate, but Zheng et al. [95] found them to be striate as well.

In conclusion, conventional multi-locus studies and genome-based phylogenetic analyses recognized separate clades for var. *arrhizus* and var. *delemar*. However, there is still zygospore formation between members of these varieties, suggesting that the mating barrier is not complete yet. This is in agreement with the detection of strains with mixed ancestry, and the lack of differences in ecology, epidemiology, and distribution between the varieties. Although there are small morphological differences between var. *arrhizus* and var. *delemar*, they are not fully consistent. Considering the dynamic genomes in *R. arrhizus*, the absence of a single gene such as lactase dehydrogenase A in var. *delemar* is not sufficient for the species rank. Until we fully understand the population structure in *R. arrhizus,* we recommend that their status as varieties be maintained.

*Amylomyces rouxii* is the name of a phenotypic variant of *R. arrhizus* that is used as a starter culture for the fermentation of soybean products. It forms mainly chlamydospores instead of sporangia, and assimilates sucrose but only small amounts of glycerol. In contrast, typical predominantly sporangia-forming strains of *R. arrhizus* assimilate glycerol but hardly any sucrose [106]. However, the ITS-sequence of the ex-neotype strain of *Amylomyces rouxii* is positioned in the clade of the ex-neotype strain of *Rhizopus arrhizus* var. *arrhizus* [22,42]. As a consequence, the species is now treated as a synonym of *R. arrhizus*. Interestingly, a recent study found strains with the phenotype of *Amylomyces rouxii* that cluster in the clade of var. *delemar* [106].

## 5. Identification and Detection

Molecular species identification based on ITS-sequences is the method of choice in Mucorales, as it has been shown by numerous studies to reliably distinguish species [21,22,29,34]. Failure of the ITS region to discriminate *R. microsporus* and *R. azygosporus* can be explained by the fact that both species actually belong to the same species [44]. In the *Mucor circinelloides* relationship, protein-coding genes such as *tsr1* or *rpb1* have a much higher resolution power than ITS, but reference sequences of these genes are usually lacking [23]. A disadvantage of using ITS sequencing for identification is that ITS copies differ slightly in some taxa, such as *Absidia* or the *Rhizopus stolonifer* group, and direct sequencing becomes impossible [22]. Also of note, in the genus *Syncephalastrum* some strains have two clearly differing types of ITS-sequences [107]. Alternatively, LSU can be used for identification and has the advantage that direct sequencing is nearly always possible. To our knowledge, this region resolves all mucoralean species, but the sequence differences among the species are relatively small.

In diagnostic facilities, matrix-assisted laser desorption ionization-time-of-flight mass spectrometry (MALDI-TOF MS) is being increasingly used for the identification of filamentous fungi. In the last few years, several studies have shown the potential of this method for a fast identification of the Mucorales [108,109,110]. Using the VITEK® MS v3.0 system, a species identification rate of 86% was achieved for 118 mucoralean isolates [111]. However, only a species identification rate of 49.5% was possible using the Bruker library. The combination of the Bruker library with an in-house database allowed MALDI-TOF MS to identify 81.1% of 111 mucoralean isolates [112].

Numerous DNA-based assays have been developed to detect the aetiological agents of mucormycosis from fresh or formalin-fixed, paraffin-embedded clinical samples. The range of methods includes PCR-RFLPs [113], conventional semi-nested PCR [114,115,116], or RT-PCR [117,118,119,120,121,122,123,124,125,126], usually targeting nuclear rDNA (SSU, LSU, ITS) or less frequently cytochrome b [118,127]. A recent study used the gene of the mucoralean-specific spore coating protein homolog, *cot*H, as the target for PCR amplification [128]. In some studies, PCR amplification was combined with another method of identification, such as reverse line blot hybridization [129] or electrospray-ionization mass spectrometry [127]. Pathonostics commercially distributes the MucorGenius assay, which is a multiplex, real-time PCR assay detecting *Rhizopus* spp., *Mucor* spp., *Rhizomucor* spp., *Lichtheimia* spp., and *Cunninghamella* spp. in respiratory tract samples and biopsies. A completely different approach for Mucorales diagnostics is the detection of Mucorales-specific T cells [130].

Last but not least, species identification based on morphology combined with growth measurement at different media and temperatures is possible in taxonomically revised taxa [23,25,29,46].

## 6. Ecology and Geographic Distribution

Mucorales are often considered to be cosmopolitan saprobes. However, for most species, the data are not sufficient to accurately assess their ecological niche or geographic distribution. For example, *Actinomucor elegans* was thought to be a saprotrophic soil fungus [131] until it was shown that it efficiently infects chafer beetle [132]. Even from the existing data, it can be safely concluded that the Mucorales are ecologically highly diverse. Beside from saprobes, they contain parasites of plants (e.g., *Choanephora*), arthropods (e.g., *Sporodiniella umbellata*), mushrooms (*Dicranophora*, *Spinellus*, and *Synzygites*), and other Mucorales (*Chaetocladium*, *Lentamyces*, and *Parasitella*) [7,80]. During the study of herbarium material from the hypogeous mushroom, *Hysterangium,* zygospores of an endoparasitic *Mucor* species were found [133]. Although the endoparasites could not be cultivated from zygospores, sequence data ascribed them to two newly described *Mucor* species. Several Mucorales have been isolated from the inside of plants that did not show any symptoms and were consequently considered endophytes, e.g., *Mucor endophyticus* ([134], as *Rhizomucor endophyticus*).

The most important habitats for saprotrophic Mucorales are soil, dead plant material, and dung. The genus *Pilobolus* is obligate coprophilous that requires dung extract or hemin for growth, while other taxa grow on normal culture media, but show a distinct association to dung (e.g., *Benjaminiella, Cokeromyces*, *Dichotomocladium*, *Ellisomyces*, *Mucor flavus*, *M. mucedo*, *M. plasmaticus*, *Pilaira*, Figure 1g,h, *Thamnidium*, *Thamnostylum, Radiomyces*) [1,7,64,135]. Coprophilous taxa do not form a monophyletic group, but occur in all lineages within the Mucorales. Several species associated to dung (e.g., *Ellisomyces* or *Thamnidium*) differ strongly from their sibling taxa (*Mucor* species in these cases) by forming sporangiola on highly differentiated sporophores (Figure 1i) [22,23]. On the other hand, there are generalists such as *Mucor circinelloides* and *M. racemosus* that have been isolated from a broad range of substrates, including dung [136]. For some mucoralean species, the evidence is sufficient to call them cosmopolitan, e.g., *Rhizopus arrhizus*, *Lichtheimia corymbifera,* or *Mucor circinelloides,* but for numerous species this is not yet the case. Most of our information on the ecology and distribution of species is based on the source provided by culture collections and clinical studies. The number of studies directly addressing the natural habitats of Mucorales [132,136,137,138,139,140] is very limited. Taxa such as *Apophysomyces*, *Saksenaea,* and *Mucor circinatus* (misapplied: *Circinella simplex*) seem to show a distribution in tropical and subtropical regions. Reports of *Apophysomyces* spp. and *Saksenaea* ssp. in colder regions are related to clinical isolates and might refer to imported clinical cases. Other taxa have been isolated from restricted areas: e.g., *Lichtheimia sphaerocystis* is only known in India, *Ellisomyces anomalus* has only been found in California, despite being widely distributed there [141], and *Radiomyces* species have been only isolated from California and Mexico [142]. A better understanding of the biology of the opportunists, including their natural niches, reservoirs, dispersal, and geographic distribution, is a prerequisite for understanding the route of acquisition and consequently for the prevention of infections. Therefore, studies addressing the ecology of the Mucorales are urgently needed.

## Figures and Tables

**Table 1:** Phylogenetic studies of the Mucorales addressing more than one locus and the marker applied. Translation elongation factor-1 alpha (*tef*), largest subunit of RNA polymerase II (*rpm1*), lactate dehydrogenase B (l*dhB*), second largest subunit of RNA polymerase II (*rpb2*), TP transporter gene (*tpt*), 20S rRNA accumulation protein (*tsr1*)and minichromosome maintenance protein (*mcm7*), cyclopropane-fatty-acylphospholipid-synthase (*cfs*), histone H3 gene (*h3*), orotidine-5`-monophoshate decarboxylase (*pyrG*), interal transcribed spacer region (ITS), nuclear ribosomal large subunit (LSU), nuclear ribosomal small subunit (SSU), intergenic spacer (IGS). * Paralogous sequences detected.

Studied Taxon	rDNA	actin	*tef*	*rpb1*	*ldhB*	*rpb2*	*tpt*	RNA Helicase	*tsr1*	*mcm7*	*cfs*	*h3*	*pyrG*	Reference
*Mucorales*	LSU/SSU		x											O’Donnell et al. 2001 [14]
*Mucorales*		x	x											Voigt et al. 2001 [15]
*Mucorales*	LSU/SSU	x	x											Hoffmann et al. 2013 [17]
*Absidia koreana*	ITS/LSU/SSU	x	x											Ariyawansa et al. 2015 [27]
*Apophysomyces*	ITS/LSU											x		Bonifaz et al. 2014 [28]
*Apophysomyces*	ITS/LSU											x		Álvarez et al. 2010 [29]
*Apophysomyces*	ITS/LSU											x		Khuna et al. 2019 [30]
*Apophysomyces*													x (+ 13 other)	Prakash et al. 2017 [31]
*Cunninghamella*	ITS		x											Yu et al. [32]
*Gongronella koreana*	ITS/LSU/SSU	x	x											Ariyawansa et al. 2015 [27]
*Lichtheimia*	ITS/LSU		x											Garcia-Hermosa et al. 2009 [33]
*Lichtheimia*	ITS/LSU	x *												Alastruey-Izquierdo et al. 2010 [25]
*Mucor*	ITS/LSU/SSU			x					x	x				Hermet et al. 2012 [34]
*Mucor / Isomucor*	ITS/LSU		x *							x				de Souza et al. [21]
*Mucor circinatus* and relatives	ITS/LSU									x				Lima et al. 2017 [35]
*Mucor circinelloides* complex	ITS			x					x	x	x			Wagner et al. 2019 [36]
*Mucor irregularis*	LSU			x		x			x	x				Lu et al. 2013 [37]
*Mucor koreanus*	ITS/LSU/SSU	x	x											Li et al. 2016 [38]
*Pilaira*	ITS												x	Liu et al. 2012 [39]
*Pilaira australis*	ITS												x	Urquhart et al. 2017 [40]
*Rhizopus*	ITS	x	x		x									Abe et al. 2007 [41]
*Rhizopus*	ITS												x	Liu 2007 [42]
*Rhizopus arrhizus*	ITS				x	x	x	x						Gryganskyi 2010 [43]
*Rhizopus arrhizus*	ITS	x	x *	x										Dolatabadi et al. 2014 [26]
*Rhizopus*	IGS													Liu et al. 2007 [42]
*Rhizopus microsporus*	ITS	x	x											Dolatabadi et al. 2013 [44]
*Rhizopus stolonifer* group	LSU													Liou et al. 2007 [45]
*Saksenaea*	ITS/LSU		x											Álvarez et al. 2010 [46]
*Saksenaea*	ITS/LSU		x											Crous et al. 2016 [47]
*Saksenaea*	ITS/LSU		x											Crous et al. 2017 [48]

**Table 2:** Genera of the Mucorales, their assignment to families according Hoffmann et al. [17], the number of species included in these genera, and their clinical importance. The genus *Mycocladus* is not listed because it does not include a recent species.

Genus	Family According to Hoffmann et al. 2013	Sequences Available	Accepted Species	Clinically Relevant Species	Species Described after the Year 2000
*Absidia*	Cunninghamellaceae	yes	20	0	7
*Actinomucor*	Mucoraceae	yes	1	1	0
*Ambomucor*	Mucoraceae (?)	no	3	0	3
*Apophysomyces*	Saksenaeaceae	yes	6	4	5
*Backusella*	Backusellaceae	yes	14	0	4
*Benjaminiella*	Mucoraceae	yes	3	0	0
*Blakeslea*	Choanephoraceae	yes	2	0	0
*Chaetocladium*	Mucoraceae	yes	2	0	0
*Chlamydoabsidia*	Cunninghamellaceae	yes	1	0	0
*Choanephora*	Choanephoraceae	yes	2	0	0
*Circinella*	Lichtheimiaceae	yes	8	0	2
*Cokeromyces*	Mucoraceae	yes	1	1	0
*Cunninghamella*	Cunninghamellaceae	yes	14	4	4
*Dichotomocladium*	Lichtheimiaceae	yes	5	0	0
*Dicranophora*	Mucoraceae	yes	1	0	0
*Ellisomyces*	Mucoraceae	yes	1	0	0
*Fennellomyces*	Lichtheimiaceae	yes	4	0	0
*Gilbertella*	Choanephoraceae	yes	1	0	0
*Gongronella*	Cunninghamellaceae	yes	6	0	4
*Halteromyces*	Cunninghamellaceae	yes	1	0	0
*Helicostylum*	Mucoraceae	yes	2	0	0
*Hesseltinella*	Cunninghamellaceae	yes	1	0	0
*Hyphomucor*	Mucoraceae	yes	1	0	0
*Isomucor*	Mucoraceae	yes	1	0	1
*Kirkiana*	Mucoraceae (?)	no	1	0	1
*Kirkomyces*	Mucoraceae	yes	1	0	0
*Lentamyces*	Lentamycetaceae	yes	4	0	0
*Lichtheimia*	Lichtheimiaceae	yes	6	3	2
*Mucor*	Mucoraceae	yes	76	13	20
*Mycotypha*	Mycotyphaceae	yes	3	1	0
*Nawawiella*	Mucoraceae (?)	no	1	0	1
*Parasitella*	Mucoraceae	yes	1	0	0
*Phascolomyces*	Lichtheimiaceae	yes	1	0	1
*Phycomyces*	Phycomycetaceae	yes	3	0	0
*Pilaira*	Mucoraceae	yes	8	0	3
*Pilobolus*	Pilobolaceae	yes	7	0	0
*Pirella*	Mucoraceae	yes	2	0	0
*Poitrasia*	Choanephoraceae	yes	1	0	0
*Protomycocladus*	Syncephalastraceae	yes	1	0	0
*Radiomyces*	Radiomycetaceae	yes	3	0	0
*Rhizomucor*	Lichtheimiaceae	yes	2	2	0
*Rhizopodopsis*	Rhizopodacea (?)	no	1	0	0
*Rhizopus*	Rhizopodaceae	yes	10	4	1
*Saksenaea*	Saksenaeaceae	yes	5	4	4
*Spinellus*	Phycomycetaceae	yes	5	0	0
*Sporodiniella*	Rhizopodaceae	yes	1	0	0
*Syncephalastrum*	Syncephalastraceae	yes	2	1	0
*Syzygites*	Rhizopodaceae	yes	1	0	0
*Thamnidium*	Mucoraceae	yes	1	0	0
*Thamnostylum*	Lichtheimiaceae	yes	4	1	0
*Thermomucor*	Lichtheimiaceae	yes	1	0	0
*Tortumyces*	Mucoraceae (?)	no	2	0	2
*Utharomyces*	Pilobolaceae	yes	1	0	0
*Zychaea*	Lichtheimiaceae	yes	1	0	0
*Zygambella*	Rhizopodaceae (?)	no	1	0	1
Mucorales total			260	39	67

**Table 3:** Clinically relevant species of the Mucorales. * For *Apophysomyces elegans* no case report based on molecular identification exists. Earlier case reports for this species might in fact refer to the later described species *A. mexicanus*, *A. ossiformis*, *A. trapeziformis,* and *A. variabilis*.

Species	Previous Names/Important Synonyms	Reference
*Actinomucor elegans*		Mahmud et al. 2011 [66]
*(Apophysomyces elegans)* *		Alvarez et al. 2010 [29]
*Apophysomyces mexicanus*		Bonifaz et al. 2014 [28]
*Apophysomyces ossiformis*		Álvarez et al. 2010 [29]
*Apophysomyces trapeziformis*		Álvarez et al. 2010 [29]
*Apophysomyces variabilis*		Álvarez et al. 2010 [29]
*Cokeromyces recurvatus*		Ryan et al. 2011 [67]
*Cunninghamella bertholletiae*		Navanukroh et al. 2014 [68]
*Cunninghamella blakesleeana*		GarcíaRodríguez et al. 2012 [69]
*Cunninghamella echinulata*		Álvarez et al. 2009 [70]
*Cunninghamella elegans*		Yu et al. 2015 [32]
*Lichtheimia corymbifera*	*Absidia corymbifera, Mycocladus corymbifer*	Alastruey-Izquierdo et al. 2010 [25]
*Lichtheimia ornata*	*Absidia ornata*	Alastruey-Izquierdo et al. 2010 [25]
*Lichtheimia ramosa*	*Absidia ramosa, Mycocladus ramosus*	Alastruey-Izquierdo et al. 2010 [25]
*Mucor amphibiorum*		Walther et al. 2013 [22]
*Mucor ardhlaengiktus*	*Mucor ellipsoideus*	Álvarez et al. 2011 [71]
*Mucor circinelloides*	*Mucor circinelloides* f. *circinelloides, Rhizomucor regularior, Rhizomucor variabilis* var. *regularior*	Wagner et al. 2019 [36]
*Mucor griseocyanus*	*Mucor circinelloides* f. *griseocyanus*	Wagner et al. 2019 [36]
*Mucor indicus*		de Repentigny et al. 2008 [72]
*Mucor irregularis*	*Rhizomucor variabilis*	Lu et al. 2013 [37]
*Mucor janssenii*	*Mucor circinelloides* f. *janssenii*	Walther et al. 2013 [22]
*Mucor lusitanicus*	*Mucor circinelloides* f. *lusitanicus*	Álvarez et al. 2011 [71]
*Mucor plumbeus*		Walther et al. 2013 [22]
*Mucor racemosus*		Walther et al. 2013 [22]
*Mucor ramosissimus*		Hesseltine & Ellis 1964 [73]
*Mucor variicolumellatus*		Álvarez et al. 2011 (as *M. fragilis*) [71]
*Mucor velutinosus*		Álvarez et al. 2011 [71]
*Rhizomucor miehei*		Walther et al. 2013 [22]
*Rhizomucor pusillus*		Iwen et al. 2005 [74]
*Rhizopus arrhizus (incl. var. delemar)*	*Rhizopus oryzae*	Dolatabadi et al. 2014 [26]
*Rhizopus homothallicus*		Chakrabarti et al. 2010 [75]
*Rhizopus microsporus*	*Rhizopus microsporus* var. *azygosporus*, var. *chinensis*, var. *oligosporus*, var. *rhizopodiformis*, var. *tuberosus*	Dolatabadi et al. 2013 [44]
*Rhizopus schipperae*		Weizmann et al. 1996 [76]
*Saksenaea erythrospora*		Álvarez et al. 2010 [46]
*Saksenaea loutrophoriformis*		Crous et al. 2017 [48]
*Saksenaea trapezispora*		Álvarez et al. 2010 [46]
*Saksenaea vasiformis*		Álvarez et al. 2010 [46]
*Syncephalastrum racemosum*		Schlebusch et al. 2005 [77]
*Thamnostylum lucknowense*		Xess et al. 2012 [78]
